# A bibliometric analysis of large language models in mental health research

**DOI:** 10.3389/fpsyt.2026.1838199

**Published:** 2026-07-31

**Authors:** Mohammad Ali Hussiny, Trust Saidi, Minna Annika Pikkarainen, Parisa Gazerani

**Affiliations:** 1 Department of Computer Science, Faculty of Technology, Art and Design, Oslo Metropolitan University, Oslo, Norway; 2 Department of Product Design, Faculty of Technology, Art and Design, Oslo Metropolitan University, Oslo, Norway; 3 Department of Rehabilitation Science and Health Technology, Faculty of Health Sciences, Oslo Metropolitan University, Oslo, Norway; 4 Department of Life Sciences and Health, Faculty of Health Sciences, Oslo Metropolitan University, Oslo, Norway

**Keywords:** artificial intelligence, bibliometric analysis, collaboration networks, digital health equity, large language models, mental health

## Abstract

**Background:**

Mental health disorders constitute a critical global health challenge, accounting for nearly 30% of the worldwide non-fatal disease burden and resulting in annual productivity losses exceeding $1 trillion. Despite increased awareness and investment, significant barriers, such as limited access to care, societal stigma, and a shortage of trained professionals, continue to obstruct effective service delivery. Recently, LLMs have emerged as transformative tools in mental health research and practice, offering potential applications in diagnostics, therapeutic support, and patient engagement.

**Objectives:**

This study aims to provide comprehensive bibliometric analysis of research on LLMs in mental health from 2020 to 2025. Specifically, it examines publication growth and temporal trends to capture the evolution of scholarly interest, assesses country- and institution-level contributions to highlight geographic and organizational patterns, and analyzes the distribution of publications across key academic sources. In addition, the study explores prevailing research themes and their evolution over time through keyword analysis and conceptual mapping.

**Methods:**

To ensure comprehensive coverage of the literature, searches were conducted across seven major sources: Web of Science, Scopus, PubMed, Dimensions, OpenAlex, Lens, and the Cochrane Library. The combined datasets comprised 426 articles, which provided a foundation for subsequent analyses. A bibliometric approach was then employed to analyze the collected data, focusing on publication trends, collaboration networks, and thematic evolution within the field.

**Results:**

The analysis revealed a rapid acceleration in scholarly output, with a compound annual growth rate of 140%, driven by advancements in models such as GPT-3 and GPT-4, alongside strategic funding and industry initiatives. The geographic distribution of research demonstrated a significant imbalance, with high-income countries, particularly the United States, dominating the field, while low- and middle-income countries were underrepresented. Thematic evolution showed a transition from foundational research (2020–2022) to applied studies with emerging topics including chatbots, multilingual models, and therapeutic applications.

**Conclusions:**

The findings underscore the need for more inclusive and globally integrated research efforts to fully realize the potential of LLMs in mental health care. Future research must prioritize equity, strengthen pathways for clinical translation, and develop robust ethical and evaluation frameworks. Policymakers, funders, and clinicians must champion interdisciplinary, ethically guided research to ensure the safe, effective, and socially responsible integration of LLMs into mental health systems.

## 1 Introduction

Mental health disorders constitute a major global health challenge, accounting for nearly 30% of the worldwide non-fatal disease burden and costing the global economy over $1 trillion annually due to lost productivity (1, 2). Despite increasing awareness and investment, mental health care systems continue to grapple with persistent challenges, including inadequate access to care, societal stigma, and a shortage of trained professionals (3). In this landscape, digital innovations, particularly those powered by artificial intelligence, have emerged as promising tools to enhance mental health services.

One of the most notable advancements in mental health is the development of Large Language Models, hereafter referred to as LLMs (4–6). According to Kumar (7) and Kalyan (8), LLMs represent an advanced category of pretrained language models characterized by the expansion of model parameters, training datasets, and computational power. These models are trained on large-scale text corpora through self-supervised learning methodologies and can perform diverse natural language processing tasks such as text generation, translation, summarization, and answering questions. By being capable of understanding and generating human-like text, LLMs have facilitated diverse applications such as conversational agents and clinical decision-making tools. Recent studies have highlighted the potential of LLMs in various mental health contexts, including diagnostics, therapeutic support, and patient engagement (9, 10). For example, LLMs have been utilized to identify depressive language on social media, craft empathetic responses in therapy chatbots, and assist clinicians with tasks like documentation and triage (11).

However, despite growing interest in these applications, the field remains fragmented. A recent scoping review identified just 34 relevant studies between 2019 and 2023 that directly explored the use of LLMs in mental health care (10). While these studies report encouraging results, they also highlight critical challenges, including concerns over data quality, ethical risks, and the lack of standardized evaluation frameworks. Moreover, although advanced LLMs like GPT-4 and instruction-tuned multilingual models have demonstrated potential in affective computing tasks, their integration into clinical workflows is still in its infancy. Although several studies have assessed specific applications of LLMs in mental health, no comprehensive bibliometric analysis has yet examined the field’s evolution, key trends, and collaborative networks. Such an analysis is essential to understand the conceptual structure of the domain based on keyword co-occurrence, identify influential works and authors, and uncover underexplored areas that warrant further investigation. It is against this background that this study aims to fill this gap by conducting a bibliometric analysis of the literature on LLMs in mental health.

To address this gap, the present study provides a thorough bibliometric analysis of research on large language models (LLMs) in mental health that has been published between 2020 and 2025. Specifically, it examines publication growth and temporal trends, identifies leading countries and institutions, analyses publication distribution across major journals and sources, and explores dominant themes using keyword co-occurrence analysis, conceptual mapping, and thematic evolution. By mapping the intellectual and thematic development of this rapidly expanding research area, the study sheds light on emerging trends and directions for future research and practice at the intersection of LLMs and mental health.

## 2 Materials and methods

This study used a bibliometric approach to systematically identify, extract, and analyze scholarly articles on the use of LLMs in mental health research. This approach was designed to quantify research activity, trace temporal growth patterns, identify leading authors and institutions, and reveal thematic clusters and emerging research areas. To this end, the study mapped publication trends, assessed citation impact, and performed a keyword co-occurrence analysis. Together, these methods offer a comprehensive overview of the influence of LLMs on mental health research and practice. Recent studies have emphasized the potential of LLMs in a wide range of applications, including clinical decision support, the early detection of psychological distress, augmenting therapy, and using conversational agents to engage patients (12–15). These advancements underscore the need for a systematic assessment of the existing body of evidence. Accordingly, this study uses bibliometric methods to map the research landscape, evaluate the current state of knowledge, and identify emerging areas of research at the intersection of LLMs and mental health.

### 2.1 Data collection

The bibliographic data were collected in September 2025 using Biblioshiny, the web-based interface of the R Bibliometrix package. This tool was chosen for its ability to integrate with multiple bibliographic databases and facilitate advanced bibliometric analyses (16). To ensure comprehensive coverage of the literature, searches were performed across seven major sources: Web of Science, Scopus, PubMed, Dimensions, OpenAlex, The Lens, and the Cochrane Library, with the distribution as follows: 110 from Web of Science, 76 from PubMed, 212 from Scopus, 55 from OpenAlex, 64 from Dimensions, 35 from Cochrane Library, 333 from The Lens, and Cochrane Library (35, later excluded due to incomplete metadata).

The search query combined LLM-related and mental-health–related terms with Boolean operators, restricted to publications between 2020 and 2025:

(“large language model” OR “LLM” OR “LLMs” OR “GPT” OR “ChatGPT” OR “Bard” OR “generative AI” OR “LLM-based chatbot” OR “ artificial intelligence “ OR “AI”) AND (“mental health” OR “psychiatry” OR “psychology” OR “depression” OR “anxiety” OR “mental disorder”) AND (“diagnosis” OR “assessment” OR “screening” OR “treatment” OR “therapy” OR “trend” OR “development”).

### 2.2 Data cleaning and standardization

The Biblioshiny tool in the Bibliometrix R package was used to clean and validate the articles, and in addition to automated cleaning procedures, a structured manual review was conducted to ensure consistency and reproducibility across databases. During this process, the titles and abstracts of each extracted record were examined independently to verify their relevance to the study objectives and to identify incorrectly indexed, duplicated or irrelevant records.

The bibliometric dataset comprised English-language publications that were indexed in the selected databases between 2020 and 2025. These publications included peer-reviewed journal articles and conference proceedings, as well as a limited number of preprints that were only retained when no peer-reviewed version of the same study was available. Editorials, commentaries, book reviews, notes and other non-scholarly document types were excluded. Only publications with a direct and explicit focus on the application, evaluation, or implications of large language models in mental health contexts were eligible for inclusion; studies addressing LLMs solely from a technical or computational perspective without relevance to mental health were excluded. The workflow consisted of the following steps:

Duplicate records were identified using a hierarchical approach implemented in Bibliometrix via Biblioshiny. Records with identical DOIs were automatically removed. When DOIs were unavailable, duplicates were detected using the tool’s built-in string similarity matching of normalized titles, combined with the first author’s name and the publication year. Institutional name variations were standardized using the tool’s normalization routines, with minor manual verification. A total of 378 duplicate records were removed from the initial 885 entries.

Following deduplication, the completeness of the metadata was assessed for each record. 35 articles retrieved from the Cochrane Library were excluded due to insufficient bibliographic information. For studies indexed across multiple databases, complementary metadata were merged to maximize record completeness and improve the quality of subsequent analyses. Metadata fields, including institutional affiliations, journal titles, and country identifiers, were then standardized to reduce inconsistencies caused by variations in naming conventions. Finally, preprints were only retained when no corresponding peer-reviewed publication could be identified at the time of data collection. Where both a preprint and a peer-reviewed version were available, only the peer-reviewed article was included to avoid duplication and potential citation bias. Retained preprints were appropriately identified during analysis.

### 2.3 Screening

**Figure 1:**
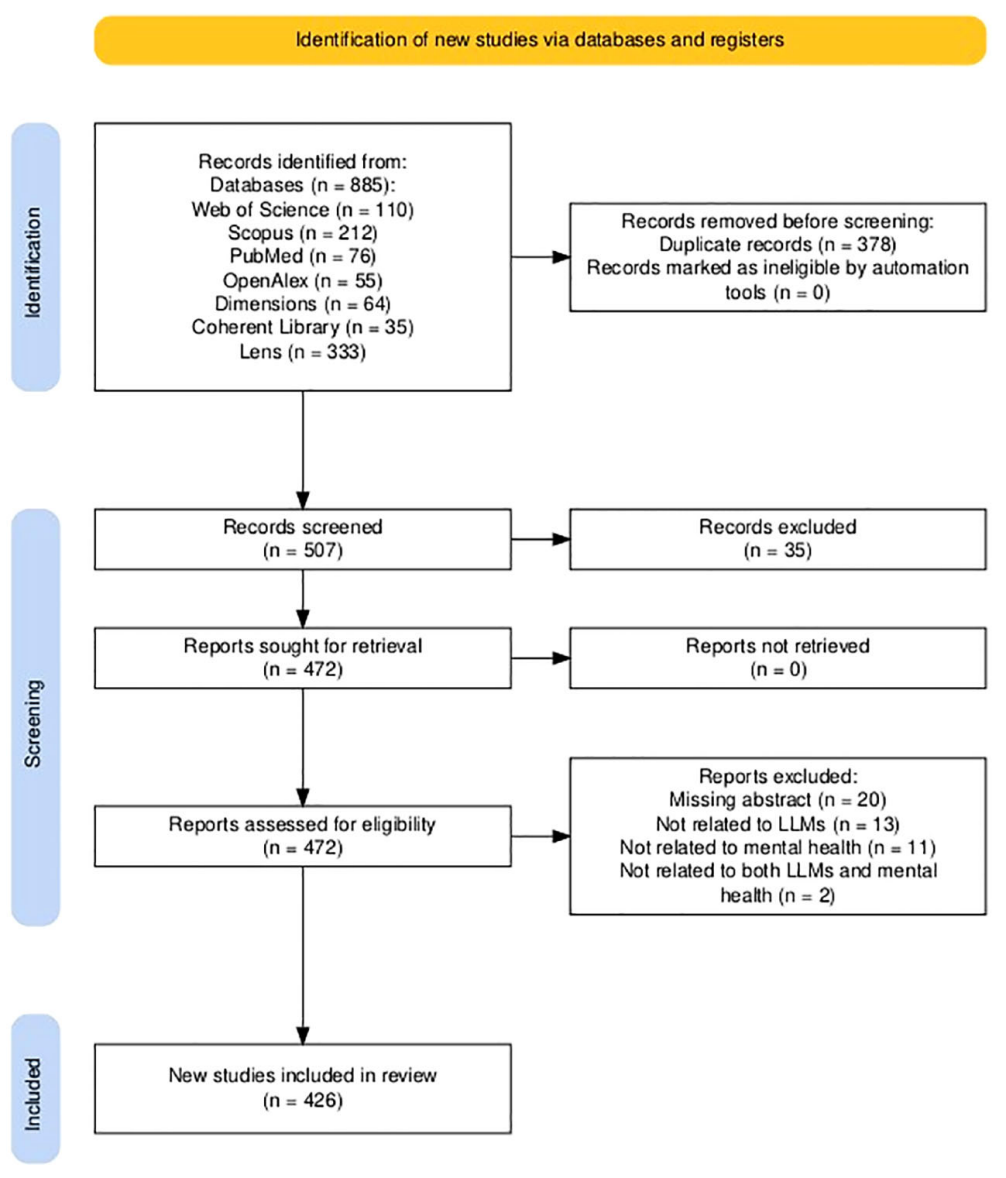
Flowchart of study identification and screening process.

Following the removal of duplicates, 507 unique records were screened; during this process, 35 records were excluded as they did not meet the inclusion criteria. This left 472 reports to be retrieved in full and assessed for eligibility, and 472 reports were successfully retrieved. During the eligibility assessment, 46 reports were excluded: 20 had missing abstracts; 13 were not related to LLMs; 11 were not related to mental health; and two were not related to either LLMs or mental health. The final analytical dataset comprised 426 documents and formed the basis for all subsequent analyses. As shown in **Figure 1**, the flowchart outlines the process of study identification and screening.

### 2.4 Metadata quality assessment

The quality of bibliographic metadata was evaluated at the article level using a revised approach designed to minimize coverage bias. Earlier drafts excluded entire journals when the majority of their metadata fields were missing. The revised protocol involved manually checking and updating individual articles with partially missing or inconsistent metadata, retrieving missing information directly from relevant databases or publisher sources. This process ensured a complete and more reliable dataset, thereby improving the accuracy and interpretability of subsequent bibliometric analyses.

### 2.5 Units of analysis and indicators

While the article focuses on the unit of analysis, additional evaluations were conducted at the country, institutional, topic, and source levels. To capture the multifaceted nature of research activity, bibliometric indicators were organized into four complementary categories. Productivity indicators were used to assess publication output, annual growth patterns, and authorship trends. Impact indicators were applied to evaluate citation performance, including citation counts, yearly citation averages, h-index and g-index measures, as well as documents with a high number of citations worldwide. Collaboration indicators assessed co-authorship relationships among authors, institutions and countries, characterizing scientific collaboration structures. Finally, conceptual indicators examined the intellectual and thematic landscape of the field through keyword co-occurrence analysis, thematic evolution and conceptual mapping.

To further examine the structural relationships within the literature, collaboration and conceptual networks were constructed. Co-authorship networks were developed at the author, institutional, and country levels, with link weights determined by the frequency of joint publications. Keyword co-occurrence networks were generated using the Louvain clustering algorithm, and association strength normalization was applied to refine the strength of associations between terms.

## 3 Results

This section presents the results of a bibliometric analysis of 426 documents that address LLMs in the context of mental health. These documents were published between 2020 and 2025. In line with the analytical framework outlined in the ‘Methodology’ section, the findings are organized according to the dimensions of productivity, impact, collaboration and conceptual structure. The results reveal trends in publication growth, influential contributors and sources, patterns of scientific collaboration and the evolution of research themes within the field.

### 3.1 Annual growth of publications

**Figure 2:**
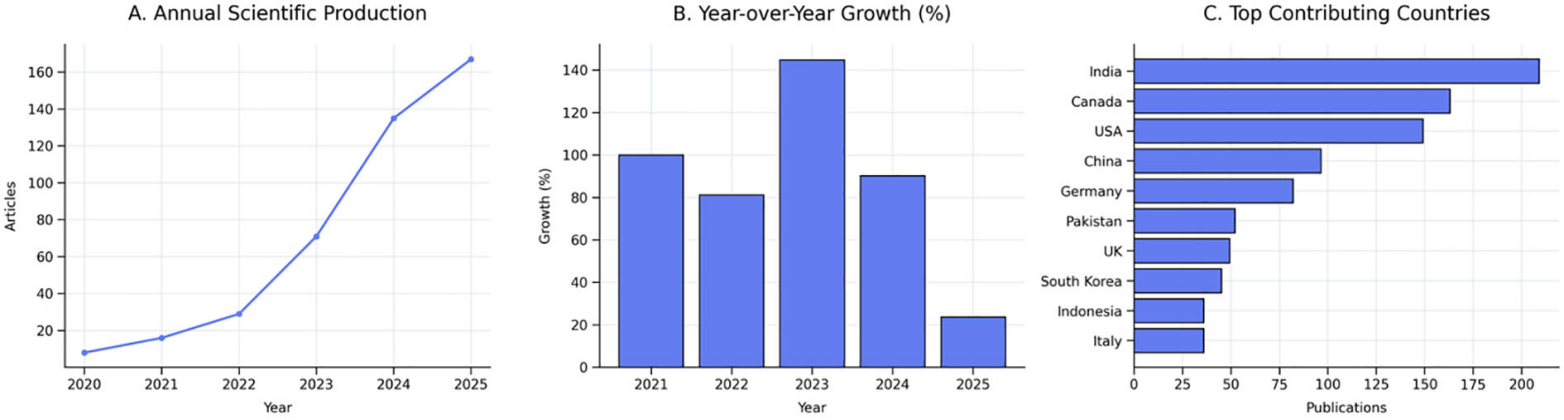
Multi-panel visualization of annual scientific production and country-level contributions.

Between 2020 and 2025, publications on LLMs in mental health increased sharply from 8 to 167 articles (**Figure 2**), indicating rapid expansion of the field. The most significant surge occurred between 2022 and 2023, with annual growth exceeding 140%. This reflects a transition from the early stages of development to accelerated adoption. This period coincided with significant advancements in LLMs, including the release of GPT-3 in 2020 and GPT-4 in 2023, as well as increased investment in AI-driven healthcare applications. While the overall trajectory shows strong momentum, the decline in growth rate after 2024 suggests the start of a stabilization phase. Notably, a substantial proportion of publications are concentrated in the most recent years, confirming that the field is still in its early stages but maturing rapidly.

### 3.2 Country contributions

Research output is highly concentrated, with the USA (315 publications) leading, followed by India (219), China (193), Canada (186), and the UK (153). While an imbalance highlights a clear discrepancy between productivity and impact. At the regional level, Asia dominates, accounting for around 55–60% of publications, followed by Europe and the Americas. Meanwhile, Africa remains marginal. Similarly, high-income countries account for the majority of publications and exert the greatest influence. Overall, these patterns reveal a field dominated by a small number of powerful research hubs, with persistent global inequalities in both contribution and impact.

**Figure 3:**
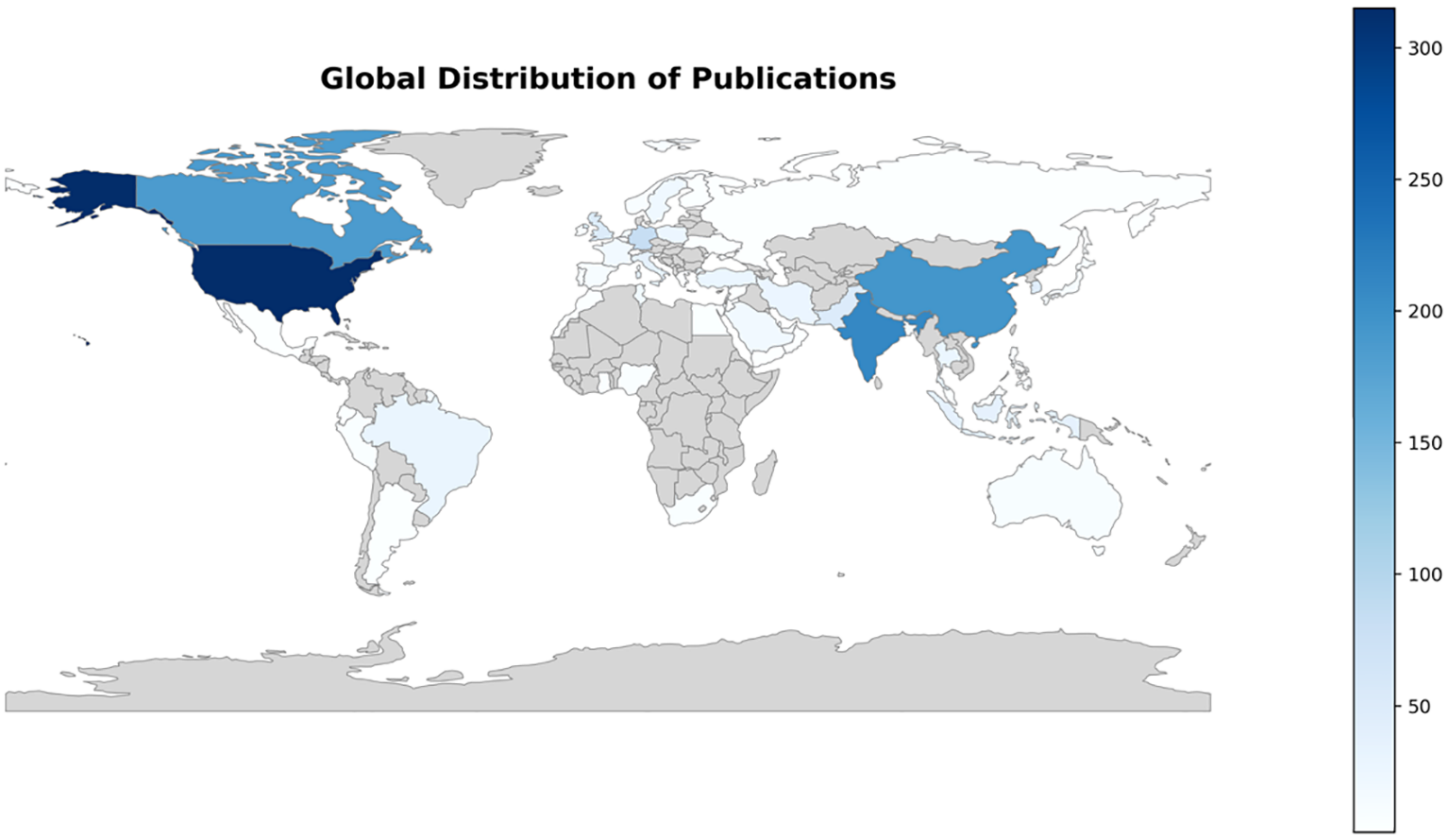
Country contributions.

**Table 1** summarizes publication characteristics by geographic region, income level, and citation impact, while **Figure 3** presents country contributions.

**Table 1:** Article characterization by region, income level, and citation impact.

Dimension	Category	Articles	Avg citations
Geographic Region	Asia	241	High
	Europe	98	High
	Americas	74	Moderate
	Africa	13	Low
Country Income	High-income	258	High
	Upper-middle	117	Moderate
	Lower-middle	46	Low
	Low-income	5	Very low
Top Contributing Countries	USA	315	Very high
	India	219	Moderate
	China	193	Moderate

### 3.3 Institutional leadership

**Figure 4:**
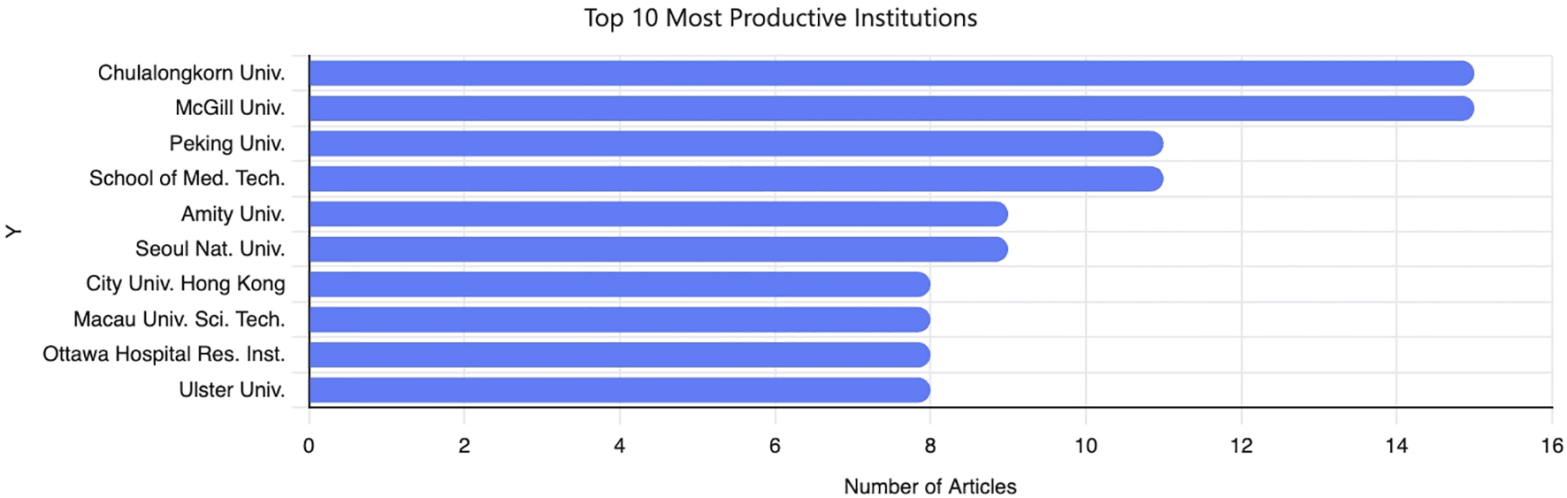
Most relevant affiliations.

Institutional contributions were assessed after affiliation names were standardized to ensure consistency. The most productive institutions were Chulalongkorn University and McGill University, which produced 15 publications each. These were followed by Peking University and the School of Medical Technology, which produced 11 publications each, and Amity University and Seoul National University, which produced nine publications each. Additional contributors, such as the City University of Hong Kong and the Ottawa Hospital Research Institute, produced eight publications each. Overall, the findings suggest a decentralized research landscape, with contributions spread across various institutions rather than being dominated by one leading organization, and **Figure 4** displays the most relevant affiliations.

### 3.4 Journals and sources

**Figure 5:**
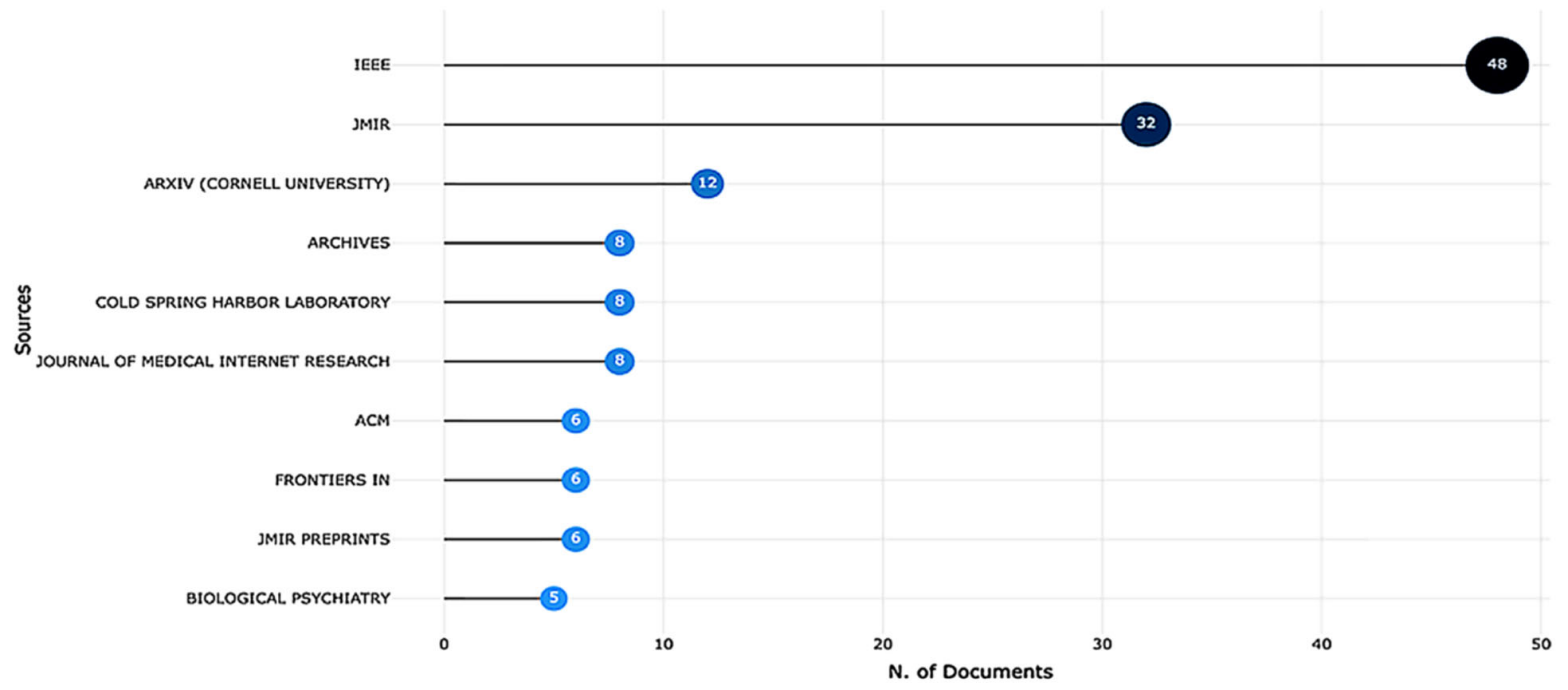
Leading publication sources. Distribution of publications across journals, conference proceedings, and preprint repositories represented in the dataset.

Research dissemination is concentrated in a few influential publications. IEEE and the Journal of Medical Internet Research (JMIR) are the most prominent sources, reflecting their key position in publishing cutting-edge work at the intersection of artificial intelligence and health. Preprint platforms such as Arxiv and institutional outlets such as the Cold Spring Harbor Laboratory also feature prominently, highlighting the rapid pace of the field and its reliance on early dissemination. These preprint records were only retained when no corresponding peer-reviewed publication could be identified during the data collection and screening process. Other notable contributors include the Archives, the Association for Computing Machinery (ACM), Frontiers, and JMIR Preprints, which together highlight the diversity of the publishing ecosystem, spanning established journals, interdisciplinary venues, and preprint servers. **Figure 5** shows the article Journals and Sources.

### 3.5 Keyword analysis and emerging themes

**Figure 6:**
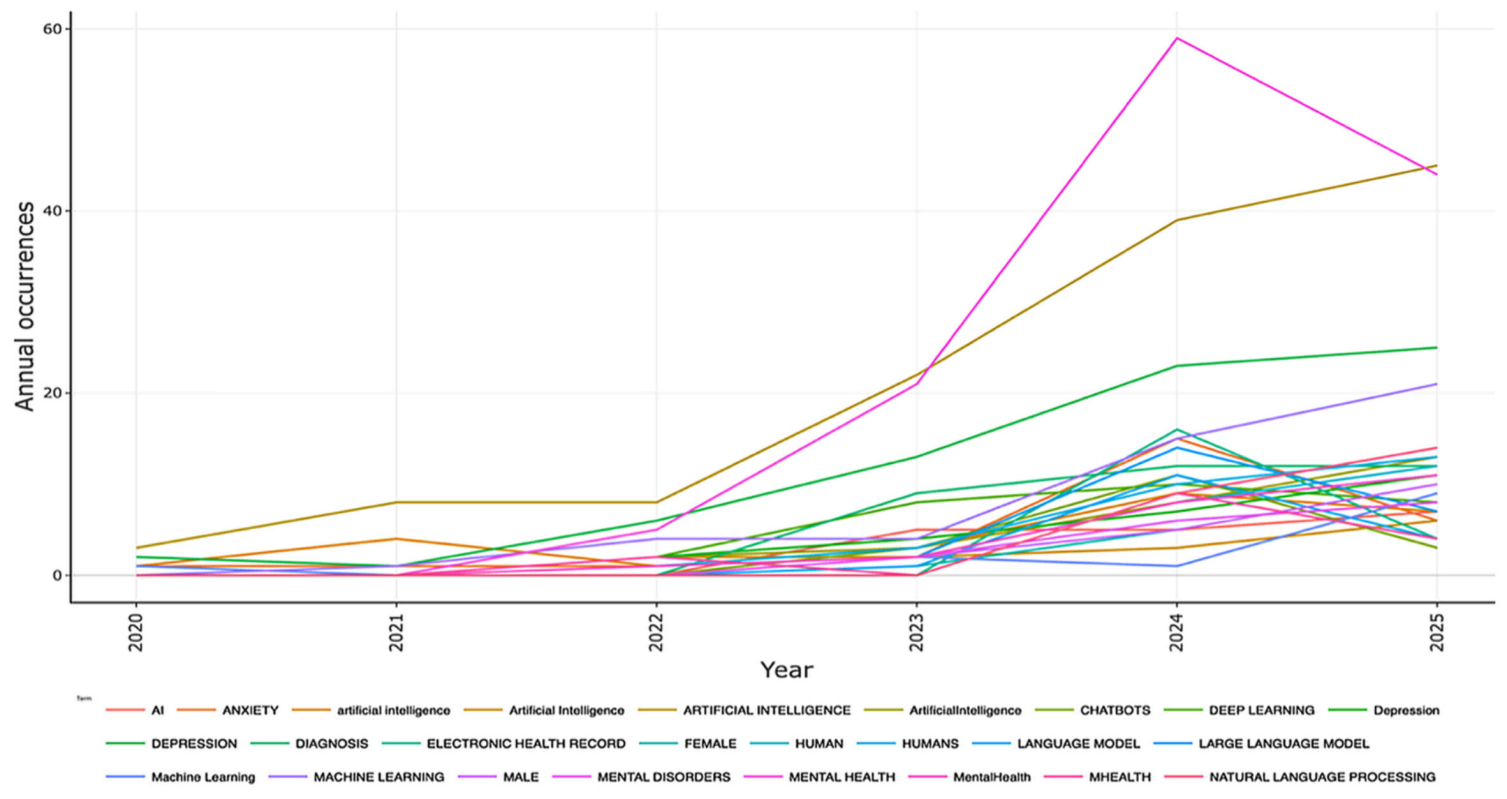
Temporal evolution of research keywords. Changes in keyword occurrence from 2020 to 2025, illustrating the pattern of major research themes over time.

**Figure 6** illustrates the temporal evolution of keyword occurrences between 2020 and 2025, highlighting a clear increase in research activity over time. During the initial period (2020–2022), keyword frequencies remained low, reflecting a conceptual focus. A transition phase is then observed between 2022 and 2023, during which time there is a gradual rise in clinically oriented terms such as ‘diagnosis’ and ‘depression’. The most significant growth occurs in 2024–2025, with mental health and artificial intelligence emerging as dominant topics, alongside others including chatbots and natural language processing. Overall, the data supports the idea of a three-phase developmental trajectory, moving from conceptual exploration to clinically oriented, application-driven research.

### 3.6 Conceptual framework

**Figure 7:**
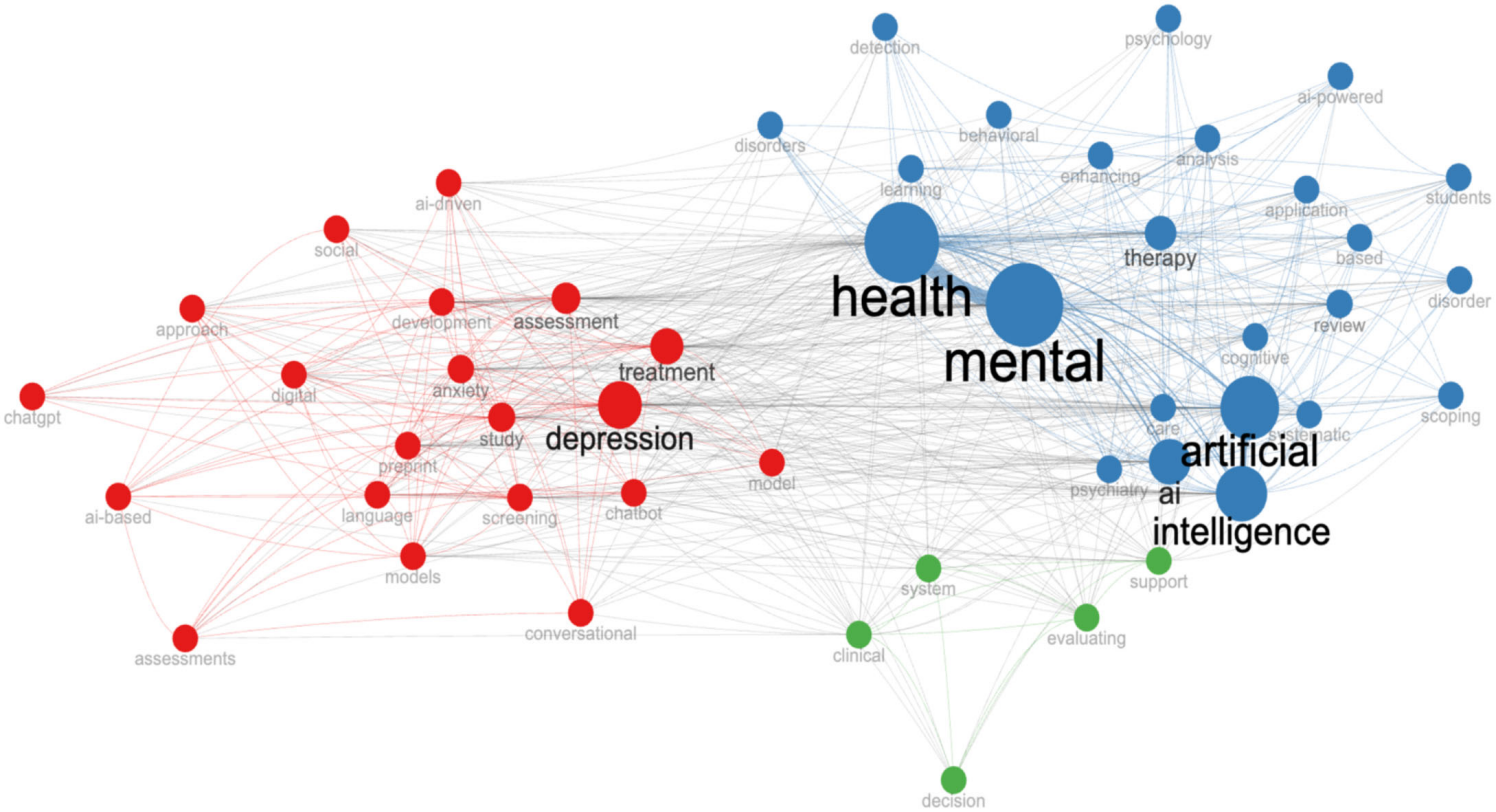
Conceptual structure of the research field. Network visualization of keyword relationships generated from co-occurrence analysis. Node size reflects keyword frequency, links represent co-occurrence strength, and colors indicate thematic clusters identified using the Louvain clustering algorithm.

The conceptual map (**Figure 7**) illustrates that the nodes represent frequently occurring keywords, with the size of the nodes proportional to the frequency of the keywords and the connecting lines indicating co-occurrence relationships. The Louvain clustering algorithm identifies thematic clusters, which are denoted by node colors: blue for technological and mental health foundations, red for clinical applications, and green for decision support and applied implementation themes.

**Figure 8:**
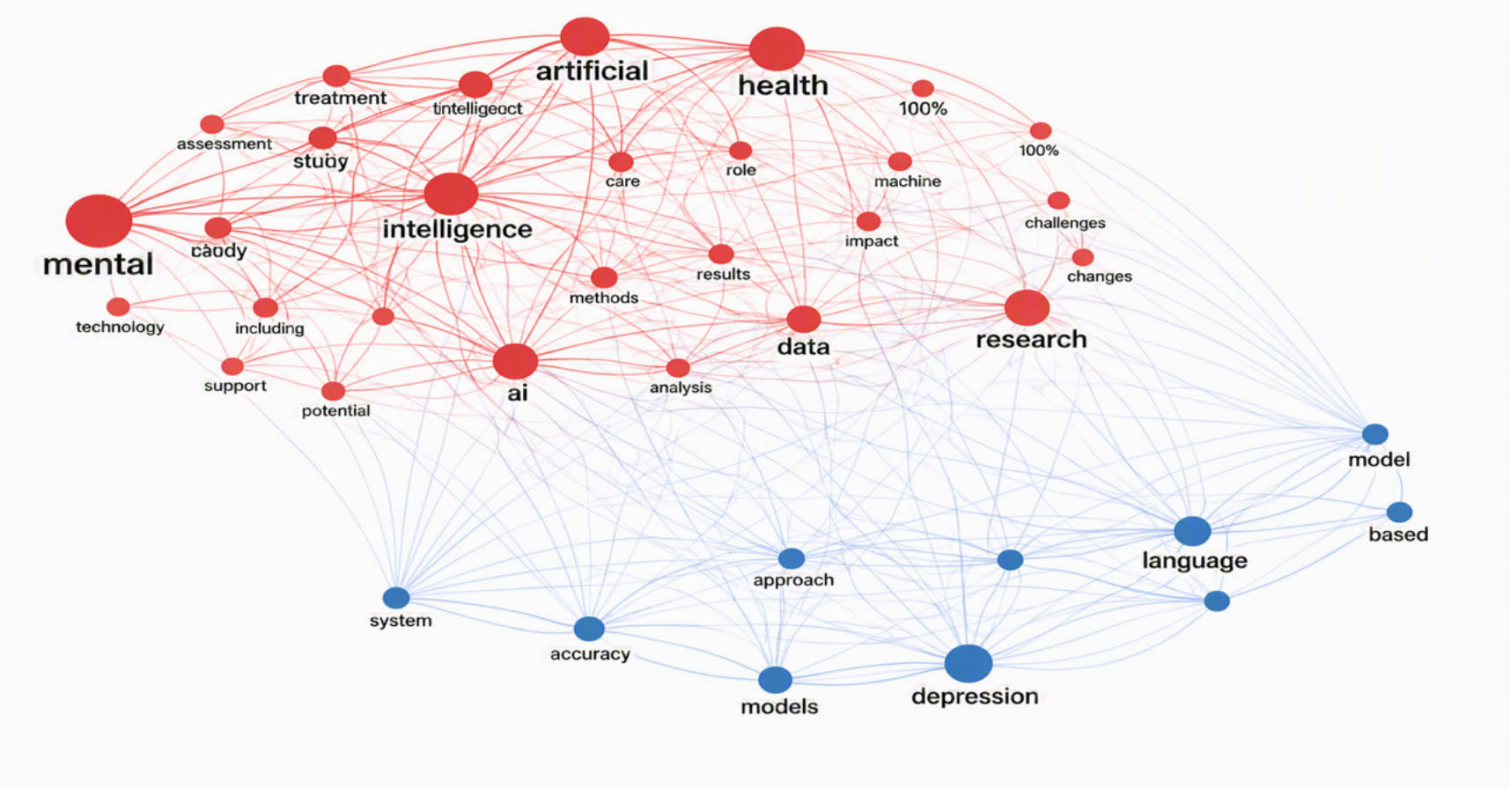
Keyword co-occurrence network. Relationships among frequently occurring keywords. Larger nodes indicate higher occurrence frequency, thicker links indicate stronger co-occurrence, and colors represent thematic communities detected using the Louvain clustering algorithm.

**Figure 8** presents a keyword co-occurrence network. The nodes represent keywords, with node size reflecting occurrence frequency and links indicating co-occurrence strength. Colors identify thematic communities, which are detected using the Louvain clustering algorithm. Blue represents technological foundations, red represents clinically oriented topics, and green represents applied decision support and implementation research.

**Figure 9:**
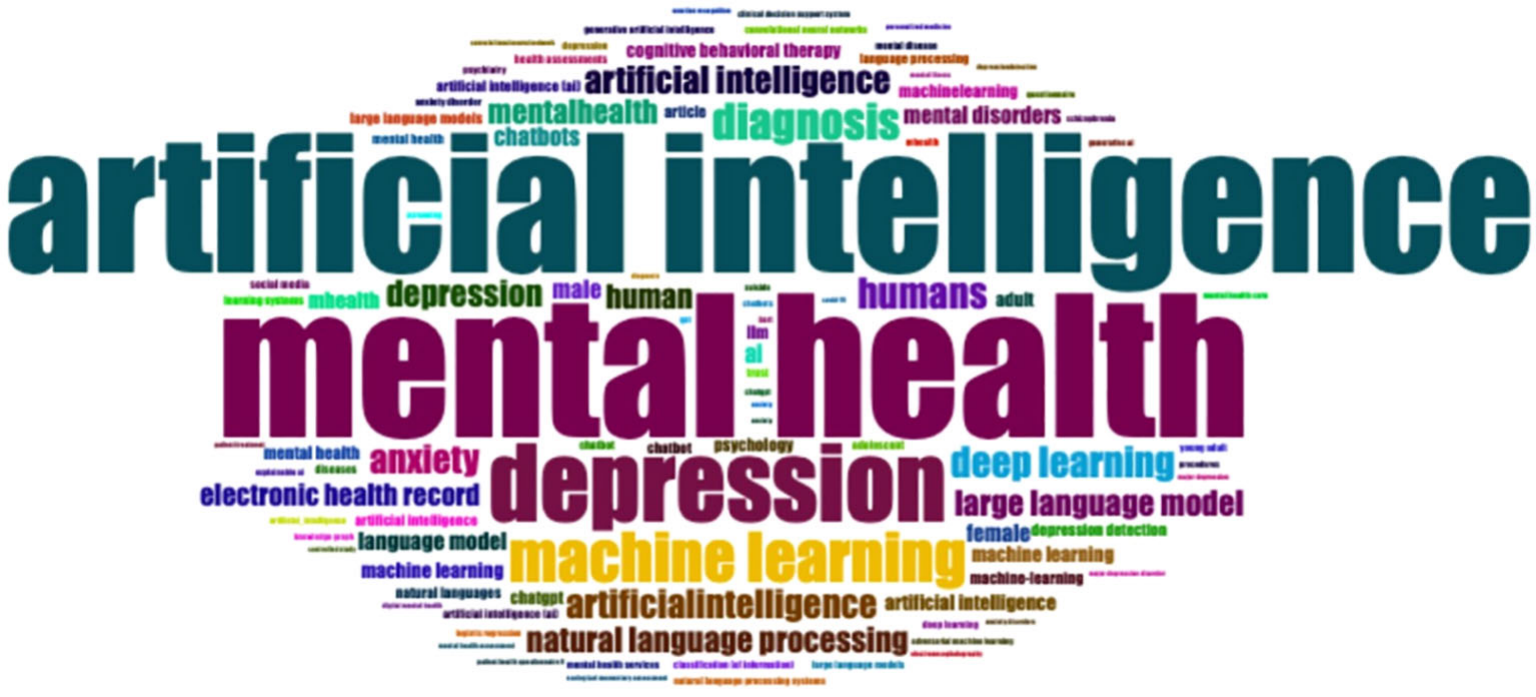
Word cloud graphic containing terms related to artificial intelligence and mental health, with prominent words including artificial intelligence, mental health, depression, machine learning, anxiety, diagnosis, deep learning, and electronic health record.

The word cloud (**Figure 9**) reinforces the dominance of mental health and artificial intelligence as central concepts. Surrounding these anchors are clinical terms such as depression, anxiety, and diagnosis, along with methodological keywords like machine learning and natural language processing. The presence of more minor yet significant terms, including chatbot, deep learning, and electronic health records, points to the integration of LLM research into broader digital health infrastructures and applied clinical contexts.

## 4 Discussion

The findings of this bibliometric analysis highlight the rapid growth and evolving trends in research on LLMs in mental health, while also exposing critical gaps in clinical translation, equity, and ethical considerations. From the analysis, it emerged that collaboration networks remain regionally concentrated, with limited engagement from low- and middle-income countries. Conceptual mapping shows a thematic shift from foundational AI research to applied clinical studies, including chatbots and therapy tools. Despite this evolution, significant gaps persist in clinical translation, ethical governance, and equitable access, which are indicated by the distribution and focus of the literature rather than directly demonstrated causal relationships, underscoring the need for inclusive, interdisciplinary, and ethically guided research to realize the potential of LLMs in mental health care fully.

By situating these findings within broader systemic contexts, including funding priorities, policy directives, and industry developments, this discussion contextualizes possible influences on observed patterns rather than attributing direct causation. The insights are critically analyzed through the lens of the research objectives. The observed trends from the findings are interpreted in light of documented developments in LLMs reported in the existing literature, which provides a plausible contextual framework for understanding shifts in research focus, rather than serving as direct explanatory evidence derived from the bibliometric analysis itself.

The surge in publications on LLMs in mental health between 2020 and 2025, reflected by a compound annual growth rate of 140%, coincided temporally with broader developments in artificial intelligence, including the release of GPT-3 (2020) and GPT-4 (2023), increasing policy attention to AI in healthcare, and expanding industry investment in LLM technologies (4, 17). This explosion in research may align with funding priorities set by prominent agencies like the National Institutes of Health, which has prioritized the integration of artificial intelligence into healthcare through programs such as its BRAIN Initiative (18). Similarly, Europe’s Horizon Europe Digital Health Strategy, with its emphasis on AI-driven medical innovation, could have contributed to the growth in publications as it spurred institutional investments in AI for mental health (19). Industry drivers can be situated alongside these factors as part of a broader set of influences. Companies like OpenAI, Google DeepMind, and Microsoft have not only released cutting-edge LLMs but have also actively supported research ecosystems by providing open-access tools and cloud infrastructure, which may have facilitated increases in research activity (6).

The momentum in terms of publications is primarily concentrated in high-income countries, with the USA leading in research output (315 publications), followed by other resource-rich nations like India, China, and the UK. This geographic imbalance may reflect broader structural inequities, particularly disparities in funding, technical expertise, and computational resources (5, 20). The underrepresentation of low- and middle-income countries in LLM research is particularly concerning, given their disproportionate share of the global mental health burden (1). Despite the potential of multilingual and low-resource LLMs to address these disparities, few studies have prioritized these applications. For instance, while recent models like GPT-4 demonstrate improved multilingual capabilities, most LLMs are optimized for English and remain ill-equipped to handle low-resource languages (21, 22). Addressing this inequity is crucial and may require targeted funding mechanisms, such as grants aimed at fostering LLM research in LMICs and initiatives to develop culturally and linguistically inclusive models.

The bibliometric analysis identifies leading contributors such as King’s College London, Heidelberg University, and the University of Cambridge, emphasizing the dominance of well-resourced universities situated in high-income countries. With access to robust funding, interdisciplinary expertise, and state-of-the-art computational infrastructure, these institutions appear prominently positioned within the collaboration networks identified, although this does not directly establish causal influence on research outcomes at the intersection of AI and mental health (4, 23). Journals such as IEEE and the Journal of Medical Internet Research (JMIR) emerge as prominent dissemination platforms, reflecting their critical role in shaping discourse at the intersection of artificial intelligence and healthcare. However, collaboration networks remain relatively siloed, with limited cross-regional partnerships. Most research collaborations are concentrated within national or regional boundaries, limiting opportunities for knowledge exchange between high- and low-resource settings. Industry-academic partnerships are reflected in the dataset primarily through publications with technical emphasis, rather than indicating the full extent of applied or clinical collaboration. For instance, much of the work funded or supported by OpenAI and Google DeepMind focuses on technical enhancements to LLMs rather than their clinical translation (6, 24). This suggests a potential need for concerted efforts to expand collaborative networks through interdisciplinary and international partnerships, especially those involving LMICs.

The thematic evolution identified through keyword analysis suggests a gradual shift from foundational research toward more applied studies. Early research (2020–2022) emphasized generic AI-related terms such as “artificial intelligence systems,” “machine learning,” and “trust,” reflecting a focus on the conceptual underpinnings of LLMs and their relevance to mental health (25). These studies often explored theoretical possibilities of LLMs for mental health diagnostics and therapy without necessarily engaging with clinical contexts. However, between 2022 and 2023, the focus shifted toward more applied language, including ‘screening,’ ‘depression,’ ‘anxiety disorders,’ and ‘psychiatry,’ alongside growing interest in the use of LLMs for specific mental health applications (4).

By 2024–2025, keywords such as “chatbot,” “therapy,” and “eating disorders” emerged, signaling a growing interest in clinically specific applications and translational research. Despite this apparent progression, the field remains disproportionately focused on conceptual and preclinical research, with limited studies progressing to real-world implementation. For instance, while LLMs have demonstrated potential in simulating empathetic responses, aiding in triage, and identifying depressive language on social media (11); however, there is a notable lack of large-scale clinical trials to validate their effectiveness in real-world mental health care settings. This translational gap is discussed in the literature as being potentially associated with multiple factors, including the high costs and regulatory complexities of clinical trials, concerns about data privacy and ethical risks, and the inherent unpredictability of LLM-generated outputs in high-stakes clinical environments (4). Moreover, while the emergence of terms such as “chatbot” and “natural language processing” reflects growing interest in user-facing applications, real-world adoption remains limited due to issues of trust, explainability, and ethical transparency. Studies have flagged concerns over LLMs’ susceptibility to generating biased or harmful responses, particularly in sensitive contexts like mental health (6). These risks, coupled with the absence of standardized evaluation frameworks, are situated within broader challenges surrounding the integration of LLMs into clinical workflows (17, 26).

The bibliometric analysis highlights inequities in research output between high- and low- to middle-income countries, reflecting underlying structural and systemic disparities beyond geographic differences. A critical barrier to equitable impact is suggested in the literature to relate to the technological architecture of current LLMs, which are predominantly trained on data derived from high-resource languages and cultural contexts. Even multilingual models, such as GPT-4, often exhibit suboptimal performance in low-resource languages, resulting in limited applicability for populations that are already underserved in mental health care delivery (27). This limitation may also reflect broader systemic conditions; it is also embedded within broader systemic conditions in which research priorities are shaped by resource availability, market incentives, and geopolitical influences which could lead to exclusion of other countries. One contributing factor discussed in prior studies is the limited availability exclusion is closely linked to the limited availability of high-quality, representative datasets in low-resource languages and underreprsented cultural contexts. LLMs rely heavily on large-scale text for pretraining, and the availability of such corpora is disproportionately skewed toward major languages like English, Mandarin, and Spanish. For LMIC contexts, where indigenous and local languages are widely spoken, the absence of annotated datasets can severely limit the relevance and reliability of LLM-generated outputs (10, 11). Even when multilingual LLMs attempt to incorporate low-resource languages, they often inherit biases from training data, exacerbating the risk of stigmatization or misdiagnosis in mental health applications (11).

Based on the findings of this study and supported by wider literature, several recommendations for advancing the safe and equitable integration of LLMs into mental healthcare emerge. Future research should prioritize rigorous clinical trials to evaluate the efficacy, safety and real-world applicability of LLM-based interventions in various mental health contexts. Additionally, multilingual and culturally sensitive models should be developed to reduce global inequities and improve accessibility for underserved populations. Standardized evaluation frameworks are also required to assess safety, effectiveness and ethical considerations, thereby strengthening accountability and transparency. Also, expanding international collaborations, particularly with low- and middle-income countries, is essential to promote inclusive innovation and equitable participation in LLM-based mental health research. This study has conducted a bibliometric analysis on the application of LLMs in the field of mental health. To build on these findings, it is recommended that future research undertake a systematic review of the literature on this topic. Such a review would offer more profound insights into how LLMs are being practically applied in mental health contexts, including their effectiveness, ethical considerations, and potential challenges. This would complement the bibliometric perspective with a more nuanced understanding of the content and quality of existing research.

## 5 Limitations

Several limitations should be acknowledged. First, the analysis was restricted to databases compatible with Biblioshiny. Thus, some relevant studies which could have been indexed elsewhere or published in non-English languages may not have been included. Although the search strategy combined LLM-specific and broader artificial intelligence terms to maximize recall, this approach may have retrieved records not exclusively focused on LLMs, despite subsequent screening. Additionally, metadata quality varied across databases. In some instances, missing details such as subject categories, corresponding author information, and cited references may have affected the analysis’s completeness. Because the final dataset included a limited number of non-peer-reviewed preprints when no published version was available, citation patterns and temporal trends should be interpreted cautiously, as preprints may undergo substantial revision following peer review, and the stability of findings over time. Citation-based indicators are time-sensitive and vary across databases, which limits cross-source comparability. Additionally, the outcomes of keyword co-occurrence, clustering, and topic modeling depend on parameter choices. Thus, alternative thresholds or algorithms could produce different thematic structures. Finally, this study does not assess the clinical effectiveness or real-world impact of LLM applications, a critical area for future investigation.

## 6 Conclusion

The intersection of large language models and mental health care represents a transformative frontier, with immense potential to address critical gaps in access, scalability, and personalization of mental health services. However, this bibliometric analysis reveals that while the field has experienced exponential growth, it remains characterized by significant inequities and limited translation into real-world applications. In addition, there is a translational gap between conceptual research and real-world clinical applications. While LLMs have shown promise in tasks like diagnostics, triage, and therapeutic support, their integration into clinical workflows remains limited. This limited clinical translation may reflect broader barriers such as the cost of clinical trials, regulatory complexities, and concerns related to trust, accountability, and data governance. This implies that for LLMs to move beyond academic curiosity and prove their utility in real-world settings, stakeholders must prioritize translational research that evaluates their safety, efficacy, and ethical implications in diverse clinical contexts. The process demands a shift in responsibility, as researchers, clinicians, and policymakers must now address the real-world complexities of implementation, including interoperability with existing health systems, the need for culturally sensitive interventions, and the sustainability of LLM-based solutions. The responsible integration of LLMs into mental health care requires policymakers, funding bodies, and clinical practitioners to focus on advancing translational research that is both ethically sound and tailored to real-world contexts. Key challenges such as bias, interpretability, and data privacy must be actively addressed, alongside efforts to promote innovation that is inclusive and aligned with both clinical priorities and societal values. These measures are crucial to ensuring the safe, equitable, and effective application of LLMs in the sensitive field of mental health care.

## Data Availability

The raw data supporting the conclusions of this article will be made available by the authors, without undue reservation.
